# Editorial: Effective occupational health and safety management in advancing global agri-food sustainability

**DOI:** 10.3389/fpubh.2024.1438907

**Published:** 2024-07-12

**Authors:** Shuva Bhowmik, Abdullah-Al Mamun, Noordiana Nordin

**Affiliations:** ^1^Centre for Bioengineering and Nanomedicine, Faculty of Dentistry, Division of Health Sciences, University of Otago, Dunedin, New Zealand; ^2^Department of Food Science, University of Otago, Division of Sciences, Dunedin, New Zealand; ^3^Department of Fisheries and Marine Science, Noakhali Science and Technology University, Noakhali, Bangladesh; ^4^Laboratory of Food Safety and Food Integrity, Institute of Tropical Agriculture and Food Security, Universiti Putra Malaysia, Selangor, Malaysia

**Keywords:** occupational health, workers' health safety, pesticides/chemicals, personal protective equipment, safety accidents, agri-food sustainability

An ideal industrial strategy for promoting and ensuring a safe and healthy work environment is effective occupational health and safety management. Global organizations are concentrating on workplace safety to reduce detrimental health effects, economics, society, environment, corporate reputation, and desired productivity in order to achieve sustainability in the agricultural, food, and fisheries sectors. Nevertheless, the high cost of training supplies, personal protective equipment (PPE), professional trainers, and activities means that the use of these efficient procedures in industrial sectors around the globe is still limited. As a result, employees experience stress and danger at work, leading to lower food productivity. Therefore, this Research Topic on “*Effective occupational health and safety management in advancing global agri-food sustainability*” addresses the implications of occupational health and safety management in advancing global agri-food sustainability, specifically in relation to the United Nations Sustainable Development Goals (UNSDGs). The Research Topic also addresses the factors that influence the level of pesticides/chemicals exposure among agricultural/industrial workers, as well as the impact of their knowledge, attitude, and practices regarding personal protective equipment. The World Health Organization (WHO) reported that workers who come into contact with pesticides at work or in their homes are most at risk for health problems. Hence, the Food and Agriculture Organization of the United Nations (FAO) and WHO jointly developed the international code of conduct on pesticide management to minimize workers' health effects and improve their knowledge, attitude, and practices regarding PPE. They also reported that for successful application of PPE during chemicals/pesticide use, it must include the following features: a face shield, well-maintained respirators with extra cartridges, eye and facial protection, washable hats, chemical-resistant boots, gloves, and aprons. The Research Topic also covers the etiology and conditions surrounding occupational accidents in different industrial sectors.

This Research Topic contains five research articles that are relevant to the present study subject. The first article, “*Exposure to organophosphate insecticides, inappropriate personal protective equipment use, and cognitive performance among pesticide applicators*”, written by Chittrakul et al., from the Department of Community Medicine, Faculty of Medicine, Chiang Mai University, Chiang Mai, Thailand. The second manuscript, “*The impact of the use of personal-protective-equipment on the minimization of effects of exposure to pesticides among farm-workers in India*”, contributed by Lari et al., from the ICMR-National Institute of Nutrition, Hyderabad, Telangana, India. The third research paper, “*Generation paths of major production safety accidents—A fuzzy-set qualitative comparative analysis based on Chinese data*”, was written by Zhou et al., from the School of Public Administration, Central South University, Changsha, Hunan, China. The fourth research manuscript, “*Causes and circumstances of accidents at work in the European Union, Slovakia and Czech Republic*”, contributed by Hollá et al., from the Faculty of Security Engineering, University of Zilina, Zilina, Slovakia and Occupational Safety Research Institute, Prague, Czechia. The fifth manuscript, “*Evaluation of the effect of photoplethysmograms on workers' exposure to methyl bromide using second derivative*”, written by Choi et al., from the Human Anti-Aging Standards Research Institute, Uiryeong-gun, Gyeongsangnam-do, Republic of Korea.

The first research article, authored by Chittrakul et al., described severe concerns surrounding the potential risks associated with pesticide exposure among agricultural workers during the pesticide application process. This study indicates that inappropriate personal protective equipment (PPE) use during pesticide application was the major factor affecting urinary organophosphate (OP) metabolite levels among pesticide applicators. The levels of urinary OP metabolites were considerably elevated for longer durations after pesticide treatment. Diethylphosphate (DEP) metabolites were found to be the primary metabolites in urine samples due to their lipophilic properties, which accumulated in fatty tissues. Inadequate use of PPE has an important role in influencing the level of pesticide exposure. The primary means of exposure to very minute pesticide particles during spraying were via dermal contact and breathing. Continuous education and training on the correct use of personal protective equipment (PPE) are crucial in modifying the behavior of agricultural workers toward PPE usage. Overall, the manuscript highlighted the effective application of PPE, which could minimize the exposure rate of OP in agricultural workers.

The second article, published by Lari et al., focused on how well-functioning systematic occupational safety and health management benefits people and organizations in creating sustainable workplaces. Personal protective equipment (PPE) is widely acknowledged as an essential tool for preventing workers from exposure to various occupational hazards that may cause accidents, injuries, or diseases. This research demonstrates that agricultural workers who are exposed to pesticides exhibit hazardous pesticide-handling techniques and have an inadequate understanding of the associated risks. Consequently, these workers show elevated levels of biomarkers. Farm workers are exposed to many potential risk factors, including inadequate PPE and unsuitable clothing while handling pesticides. Additionally, they may lack a technical understanding of the dangers associated with pesticide toxicity and may exhibit unwillingness or ignorance to follow proper agricultural practices. Therefore, it is essential to provide safety training and ensure the availability of cost-effective and reusable PPE at the farming site. In summary, the research article elucidated that the use of PPE during pesticide application could minimize health effects and improve obeying good agricultural practices for agri-food sustainability.

Zhou et al. analyzed the main path of occurrence and their mechanisms during prime production safety accidents in China. The authors stated that deviant production attitudes and inadequate external supervision may hinder the management's capacity to ensure safety, leading to vital production safety accidents. The authors also summarized five conditional variable paths and their combinations, with particular emphasis on three paths: technology to management, individual to technology to management, and individual to technology to management to environment. These paths are significantly influential in causing major production safety accidents. In general, the article demonstrated the causes of accidents in the workstation and the possible mitigation to reduce accident and worker risks.

Hollá et al. reported the causes and present circumstances of accidents occurring at various workstations across the European Union (EU), Czech Republic, and Slovakia. The authors elucidated that searching for effective tools and techniques is challenging for maintaining occupational health and safety. The identified five significant categories are office work, retail, manufacturing and processing companies, constructions, and healthcare, where a considerable number of workers are involved. The authors identified three leading factors contributing to accidents in the study areas between 2010 and 2018: insufficient evaluation of risks, the absence of necessary qualifications for safe employment, and the adoption of risky work techniques and practices. Overall, the research stated that challenges/causes need to be identified to prevent worker accidents in the EU industry.

The fifth and final article on this Research Topic, published by Choi et al., evaluated the negative effect of photoplethysmograms on workers' exposure to methyl bromide using the second derivative. Due to its exceptional permeability and insecticidal properties, methyl bromide (MB) is the only fumigant that is extensively used for quarantine pre-shipment treatment. It has a critical exception for the purpose of soil fumigation. However, there has been a lack of studies investigating the impact of MB exposure on vascular health. The authors conducted measurements of urinary Br– concentrations and SDPTG (second derivative of the photoplethysmogram) indices on 44 fumigators (the study group) and 20 inspectors (the control group) both before and after fumigation. These indices are used to quantify the processes of aging and vascular burden. The findings suggested that the health of the fumigator group was adversely affected by changes in SDPTG indices and Br– levels after exposure to MB-related labor despite the absence of any noticeable symptoms. In summary, the manuscript describes workers' need to maintain proper procedures while handling chemicals to reduce adverse health effects.

The present state of industrial worker occupational health management systems, exposure to pesticides/chemicals, use of PPE, and causes of safety accidents across the global industry are highlighted in published articles on the current Research Topic, along with recommendations for suitable management strategies to enhance worker health and safety for enhancing world agri-food sustainability. In future, relevant research studies are needed to consider several factors, including demographics, industrial type, safety law, and environmental and behavioral properties of workers in a holistic approach to ensuring occupational health and safety.

The guest editors of this Research Topic would like to express their gratitude to the chief editor of the journal, the professionals responsible for managing the journal, peer reviewers, and the authors/contributors for their invaluable contributions in facilitating the creation of this publication. This Research Topic would be of great interest to the scientific community and Frontiers in Public Health broad readership.

## Author contributions

SB: Conceptualization, Investigation, Project administration, Writing – original draft, Writing – review & editing. A-AM: Conceptualization, Investigation, Writing – original draft, Writing – review & editing. NN: Conceptualization, Investigation, Writing – original draft, Writing – review & editing.

